# Cross‐cultural adaptation and measurement properties of the Persian version of the modified Cincinnati knee rating system

**DOI:** 10.1002/jeo2.70003

**Published:** 2024-08-27

**Authors:** Nasim Eshraghi, Peyman Mirghaderi, Reza Omid, Mohamad Sajadi, Amirreza Pashapour‐Yeganeh, S. M. Javad Mortazavi

**Affiliations:** ^1^ Surgical Research Society (SRS), Students’ Scientific Research Center Tehran University of Medical Sciences Tehran Iran; ^2^ Joint Reconstruction Research Center Tehran University of Medical Sciences Tehran Iran; ^3^ Vali‐E‐Asr Reproductive Health Research Center, Family Health Research Institute Tehran University of Medical Sciences Tehran Iran

**Keywords:** anterior cruciate ligament, modified Cincinnati knee rating system, Persian, questionnaire, reliability, validity

## Abstract

**Purpose:**

To validate and assess the cross‐sectional adaptation of the modified Cincinnati knee rating system (MCRKS) Persian translation.

**Methods:**

To assess test‐pretest reliability, 102 participants were asked to fill out the MCRKS (Per) scale after anterior cruciate ligament (ACL) reconstruction surgery. Internal consistency (Cronbach's *α*), reliability (intraclass correlation coefficients), construct validity (Pearson's *r*) and sensitivity (floor/ceiling effect) were determined. In addition, patients completed other relevant measures as the ACL return to sports after injury (ACL‐RSI) survey, hospital for special surgery ACL satisfaction survey (HSS ACL‐SS), visual analogue scale (VAS) of pain and patient's satisfaction, Tegner activity score (TAS), single assessment numeric evaluation, and Lysholm score.

**Results:**

Using MCRKS (Per), the internal consistency (Cronbach's *α*) was 0.9 (if item deleted: 0.81–0.86); the construct validity (Pearson's *r*) varied between –0.50 (for VAS pain scale) and 0.79 (for Lysholm score); the reliability (ICC value) varied between 0.82 and 0.97; furthermore, no ceiling or floor effect was present.

**Conclusion:**

The MCRKS (Per) has adequate measurement properties and is considered a valid, reliable and sensitive instrument which can identify clinical outcomes after ACLR surgery.

**Level of Evidence:**

Level IV.

**Abbreviations**
ACLR: anterior cruciate ligament reconstruction
ACL‐RSI: anterior cruciate ligament return to sport after injury
CKRS: Cincinnati knee rating system
COSMIN: COnsensus‐based Standards for the selection of health Measurement INstruments
HSS ACL‐SS: hospital for special surgery ACL satisfaction
ICC: intraclass correlation coefficients
IKDC: International Knee Documentation Committee
KOS: knee outcome survey
PROMs: patient‐reported outcome measures
SANE: single assessment numeric evaluation
SD: standard deviation
TAS: Tegner Activity Scale
VAS: visual analogue scale

## INTRODUCTION

The susceptibility to anterior cruciate ligament (ACL) ruptures is contingent upon a confluence of individual and environmental factors. Individual risk factors encompass the female gender [20], anatomical variations such as increased pelvic width, diminutive femoral dimensions, and heightened joint and ligamentous laxity, which collectively augment the strain exerted upon the knee joint [49]. Environmental risk factors include direct trauma to the lower extremity and participation in agility‐oriented sports necessitating rapid changes in direction, such as soccer, basketball, tennis, and lacrosse [20]. Despite the diligent endeavours of clinicians and researchers to mitigate the risk, the incidence of ACL injuries has increased twofold over the past two decades [25, 34]. Approximately 200,000–250,000 ACL injuries occur annually in the United States [35, 55], with minors under the age of 18 accounting for almost 25% of these injuries [2, 16]. For individuals who experience long‐term functional instability or recurrent instances of giving way, it is advisable to undergo anatomical ACL restoration [15].

Since its inception in 1983, the initial subjective Cincinnati knee rating system (CKRS) has been utilised extensively as a comprehensive knee assessment tool to identify ACL deficient knees [39, 40]. This scale is intended to detect clinical changes with the utmost sensitivity as it assesses the clinical condition of patients following surgical procedures [5, 44, 46]. Risberg et al., [43] examined the sensitivity of CKRS, IKDC, and Lysholm to variations over time in patients who had undergone ACLR surgery. During a two‐year follow‐up, the CKRS was the only sensitive instrument capable of identifying changes in clinical condition. Furthermore, CKRS is employed in research endeavors that assess the efficacy of various rehabilitation protocols and ACL surgery types [4, 8, 14, 21, 53, 54]. After undergoing revisions in 2000, MCRKS was founded in 1993. The patient is solely required to provide input on eight items comprising three subscales; these subscales assign an aggregate score of 6–100 (representing optimal function) and assess symptoms, function, and activities of daily living. To the best of our knowledge, the CKRS has been translated into Italian [12] and Brazilian‐Portuguese, and these translations have been confirmed to serve as a reliable foundation for comparing results across different investigations [42]. However, in the Italian version the authors cut down the original version by omitting some sections of the questionnaire [12].

Given the significant duration needed by both patients and professionals to fill out the paperwork, many writers utilise a modified version of the CKRS (MCRKS) to assess their patients and streamline data gathering [7, 31, 47]. This version refers to a widely used and not recommended short questionnaire consisting of eight items [1]. It has been proven to have a relationship with other validated surveys, such as the International Knee Documentation Committee (IKDC) subjective survey [5]. This version has only been validated and translated into Arabic [28].

The decision to culturally adapt and translate the MCRKS is driven by the need to introduce a concise Persian measurement tool for evaluating ACLR surgery and assessing its divergent and predictive validity in comparison to the original, lengthier version. This abbreviated version can be valuable in medical and rehabilitation settings with high patient volume. We anticipate that the MCRKS scale will exhibit favourable reliability, validity, and responsiveness when assessing the psychological preparedness for resuming sports activities in patients who have undergone ACL repair surgery.

## MATERIALS AND METHODS

### Study design

A cross‐sectional study was performed on adult patients who received ACLR surgery at our tertiary referral centre (Imam Khomeini Hospital, Tehran, Iran) from 2018 to 2022. In order to be eligible for this study, individuals must be above the age of 14 and have a confirmed diagnosis of a total or partial rupture of the ACL, regardless of the reason (whether it be sports‐related or not). The tear may or may not be accompanied by damage to the meniscus. Additionally, participants must have a scheduled surgery for ACL reconstruction (ACLR). All patients exhibited proficiency in all three linguistic modalities (writing, speaking, and reading) in the Persian language. Patients who were declined participation in the knee‐related test were excluded from the study. Furthermore, study participants were disqualified if they had articular cartilage repair, bilateral ACL injuries, or multiligament restoration. Prior to surgery, patients submitted written informed permission, which was authorised by our Institutional Review Board (IRB approval ID: IR.TUMS.MEDICINE.REC.1400.1462) in accordance with the Helsinki principles [19]. All patients were cognizant of the study's objective and provided verbal consent to participate in our research.

### Translation and cross‐cultural adaptation

**Figure 1:**
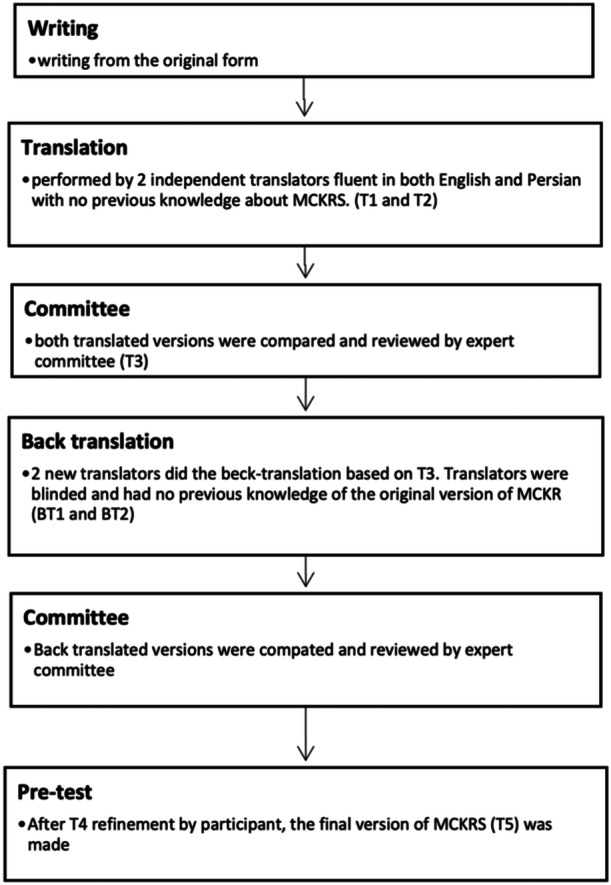
Cross‐cultural adaptation and translation of the modified Cincinnati knee rating system (MCRKS) flowchart.

The translation process was performed by two independent translator (Amir Rakhshan and Alireza Moharrami) according to proposed guideline by Guillemin et al [22] and it is presented in (Figure 1). For pretest, we asked a random group of participants (*n* = 10) to answer the prepared version of questionnaire and explore each of them about the meaning and clarity of each item. Scoring was from 0—‘I understand nothing’; 1—‘I barely know what's the meaning’; 2—‘I understand the question’. With a Total score of 20, so study participants were able to answer the items properly. We recorded any suggestions participants had regarding the structural and functional format of the scale. T4 which was the final result of committee confirmation of back translation, was then adjusted and refined according to participants' responses. Therefore, the final version (T5) was provided and ready to use (Figure 1).

### Forward translation

Two English‐Persian translators were scheduled to adapt the condensed form of the original MCRKS for use in their respective cultures. Professional translators can take cultural differences into account. The initial translator (T1) possessed a profound understanding of medical terminology, exhibited extensive knowledge in orthopaedics, and comprehended the overarching objective of the study. T2 lacked medical training and possessed no comprehension of the subject matter being translated.

### Consensus and revision

The original versions (T1 and T2) were reviewed and compared by a group consisting of three knee surgeons and two expert reviewers. According to the reviews, T2 is not much different from T1. The final version (T3) of MCRKS (Per) was a primary Persian version that was agreed upon by all parties.

### Backward translation

Two blind translators (BT1 and BT2) worked to convert the Persian original into English. Two reverse‐translated versions (BT1 and BT2) proceeded to the next phase as a consequence.

### Expert committee

The same committee meticulously assessed both the original scale and its forward and backward translated equivalents (T1, T2, BT1 and BT2). Improvements to the questionnaire were suggested and implanted. MCRKS (Per) T4, the final version, is the outcome.

### Data analysis

The data analysis was conducted using the Statistical Package for Social Studies (SPSS 23; IBM Corp). Mean ± standard deviation (SD) was used to describe continuous variables, whereas frequency was used for categorical variables. The research was conducted using the outcomes of the questionnaires administered following ACLR surgery. An analysis was conducted on the measurement properties listed below:

*Test‐retest reliability*: It is defined as two administrations of questionnaire after two weeks when targeted concept is not changed. 102 patients were included in test‐retest measurement in order to demonstrate the consistency of the results. For test‐pretest reliability, we used intraclass correlation coefficient (ICC). A correlation coefficient value over 0.40 and 0.80 as satisfactory and excellent reliability respectively [3].

*Construct validity* **:** Spearman ρ coefficient was used to assess the correlation between Persian MCRKS subscales and in total and other instruments: VAS pain, Lysholm, Hospital for Special Surgery ACL satisfaction survey (HSS ACL‐SS), ACL‐RSI, SANE and TAS. We employed the second hypothesis: The correlation between correspond domains in MCKRS and HSS ACL‐SS or ACL‐RSI should be more significant than that of other domains. The correlation shows as strong (ρ≥0.5), medium (0.3≤ρ≤0.5) and weak (ρ<0.3) [11]

*Ceiling and floor effect**:*** content validity was evaluated via ceiling and floor effect; it's done to assess sensitivity of an instrument to detect different clinical outcomes and proportion of patient who had obtained the maximum and minimum scores. Researchers had endorsed that the effect size below 15% is fair enough to prove the content validity of a questionnaire [3].

*Criterion validity*: we adopted Lysholm and VAS pain scores as gold standards to calculate the correlation strength (by Spearman ρ coefficient) of our measure with them. We considered Spearman's ρ > 0.55 (confidence interval [CI] = 95%) as an adequate cut‐off [18, 48].

### Patients and scores

In order to properly examine construct validity, reproducibility, ceiling and floor effects, and internal consistency, we needed a sample size of at least 100 patients, as suggested by Terwee et al. [36, 52]. A total of 102 patients aging 14–52 years enroled into the study (Table 1). Patients were asked to complete the validated Persian version of HSS ACL‐SS [33], Lysholm, Single Assessment Numeric evaluation [13], visual analogue scale (VAS), ACL‐RSI [17, 41], and Tegner activity scale (TAS). Negahban and others have cross‐culturally adapted the Persian version of the Tegner activity scale [38]. Informed consent was procured from all study participants. Furthermore, informed consent for the use of identifying information in an online open‐access publication was also obtained from all participants.

**Table 1:** Characteristics of the study population.

Variable (*N* = 101)	Mean ± SD or *n* (%)
Sex (male)	90 (89.1)
BMI, kg/m^2^	22.8 ± 3.4
Age, years	29.1 ± 8.0 (range: 18–52)
Side	
Left	39 (38.6)
Right	62 (61.4)
Follow‐up (years)	2.5 ± 1.2 (range: 1–6)
Mechanism	
Sport	65 (64.4)
Contact	16 (15.8)
Noncontact	49 (48.5)
Accident	20 (19.8)
Falling	12 (11.9)
Chronic	4 (4.0)
Sports before Injury	
Soccer	54 (53.5)
Other	17 (16.8)
None	30 (29.7)
High‐risk sports for ACL tear (Football, basketball, lacrosse, rugby, and skiing)	
Yes	56 (55.4)
No	15 (14.9)
None	30 (29.7)
Preinjury level of sport	
Competitive	24 (23.8)
Recreational	47 (46.5)
None	30 (29.7)
Time from injury to surgery (months)	21.9 ± 40.0 (range: 0.3–240)
Postoperative scores	
MCRKS	65.9 ± 22.6
Pain	12.0 ± 5.8
Swelling	7.6 ± 3.3
Giving away	15.1 ± 6.1
Overall activity level	12.7 ± 5.0
Walking	7.6 ± 2.5
Stairs	3.6 ± 1.3
Running activity	5.1 ± 3.6
Jumping	2.4 ± 1.6
ACL‐RSI	30.7 ± 27.1
Emotion	30.4 ± 31.3
Confidence in performance	30.8 ± 28.6
Risk appraisal	31.5 ± 30.9
Tegner	Median= 5 (range: 0–10)
Lysholm	73.7 ± 19.3
VAS pain	2.2 ± 2.7
SANE	60.4 ± 24.2
HSS ACL‐SS	27.0 ± 11.3

Abbreviations: ACL‐RSI, anterior cruciate ligament return to sport after injury; BMI, body mass index; HSS ACL‐SS, hospital for special surgery ACL satisfaction; MCRKS, modified Cincinnati knee rating system; SANE, single assessment numeric evaluation; SD, standard deviation; VAS, visual analog scale.

## RESULTS

### Patients' characteristics

A total of 102 patients with ACLR were included in the study. All of participants completed the study, therefore no data was excluded from the study. Only 55.4% (*N* = 56) patients were high risk [24, 37] for ACL tear. Study participants consist of professional athletes and members of the active population. The injury mechanism for 64.4% of patients (*N* = 65) was related to sports. Further Demographic data and clinical characteristics of the participants are presented in Table 1.

### MCRKS (Per)

The subscales of MCRKS (Per) had an internal consistency (total Cronbach's *α* = 0.908) measuring Cronbach's *α* of each eight items with the highest impact on alpha coefficient if omitted for item‐3 ‘giving away’ (*α* = 0.896) and lowest for ‘pain’ (*α* = 0.812) (Table 2).

**Table 2:** Item‐total statistics for each item of the questionnaire.

Item	Scale mean if item deleted	Scale variance if item deleted	Corrected item‐total correlation	Cronbach's *α* if item deleted
Pain	53.9604	319.710	0.765	0.812
Swelling	58.3366	420.967	0.606	0.833
Giving away	50.8713	356.945	0.510	0.861
Overall activity level	53.2079	356.818	0.684	0.821
Walking	58.2723	433.321	0.710	0.830
Stairs	62.3688	465.409	0.780	0.843
Running activity	60.8713	394.757	0.722	0.819
Jumping	63.5569	457.486	0.760	0.839

The test‐pretest intraclass correlation value (ICC) was 0.971 (*p* < 0.001) between total scores of test and retest phases. The most significant correlation was for swelling subscale (ICC = 0.829) (Table 3). All items' ICC was satisfactory and considered excellent (r≥0.81).

**Table 3:** Spearmen correlation coefficients between the test (t) and retest (rt) of the modified Cincinnati knee rating system (MCRKS) to assess the test‐retest reliability.

MCRKS subscales	Pain (rt)	Swelling (rt)	Giving away (rt)	Activity level (rt)	Walking (rt)	Stairs (rt)	Running activity (rt)	Jumping (rt)	Total score (rt)
Pain (t)									
* r*	0.944	‐	‐	‐	‐	‐	‐	‐	‐
* p*‐Value	<0.001								
Swelling (t)									
* r*	‐	0.829	‐	‐	‐	‐	‐	‐	‐
* p*‐Value		<0.001							
Giving away (t)									
* r*	‐	‐	0.962	‐	‐	‐	‐	‐	‐
* p*‐Value			<0.001						
Activity level (t)									
* r*	‐	‐	‐	0.905	‐	‐	‐	‐	‐
* p*‐Value				<0.001					
Walking (t)									
* r*	‐	‐	‐	‐	0.850	‐	‐	‐	‐
* p*‐Value					<0.001				
Stairs (t)									
* r*	‐	‐	‐	‐	‐	0.927	‐	‐	‐
* p*‐Value						<0.001			
Running activity (t)									
* r*	‐	‐	‐	‐	‐	‐	0.938	‐	‐
* p*‐Value							<0.001		
Jumping									
* r*	‐	‐	‐	‐	‐	‐	‐	0.872	‐
* p*‐Value								<0.001	
Total score (t)									
* r*	‐	‐	‐	‐	‐	‐	‐	‐	0.971
* p*‐Value									<0.001

Content validity was evaluated through the floor and ceiling effects. Five (5.0%) had a 100 score (ceiling effect). However, no one got a 0 score (floor effect). Researchers had endorsed that the effect size below 15% is fair enough to prove the content validity of a questionnaire. Therefore, the MCRKS (Per) content validity was satisfactory.

**Figure 2:**
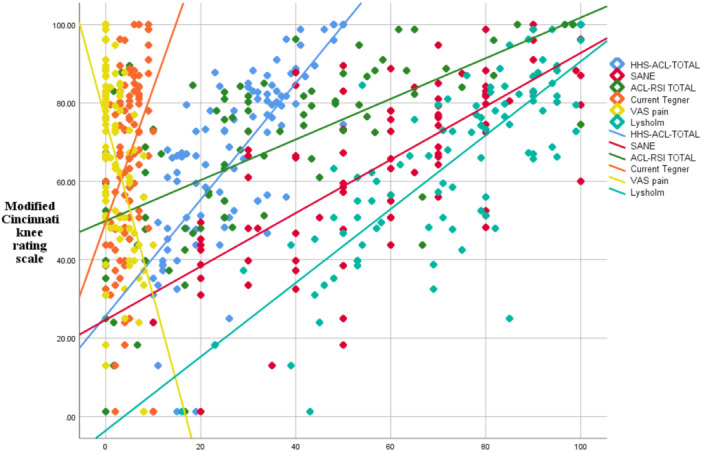
Significant correlation between the Persian modified Cincinnati knee rating system (MCRKS) score and other relevant scales.

The ACL‐RSI (*r* = 0.642), TAS (*r* = 0.421), SANE (*r* = 0.721), Lysholm (*r* = 0.794), HSS ACL‐SS (*r* = 0.782), and VAS pain ratings (*r* = –0.507) revealed significant correlations with the MCRKS score (all *p* < 0.001) (Figure 2).

Regarding the construct validity, comparing the correlation coefficients of Lysholm, VAS, and MCRKS domains indicated that there remained a stronger association between the two quite similar parts rather than irrelevant ones. VAS pain and Lysholm pain scores were more significantly correlated to the MCRKS pain (question 1) (*r* = −0.538, *r* = 0.649, *p* < 0.001) than the other sections of MCRKS (*r* < 0.647) (Table 4). Also, for MCRKS swelling domain with Lysholm swelling domain (*r* = 0.785, *p* < 0.001), and MCRKS giving away domain with Lysholm instability domain (*r* = 0.719, *p* < 0.001) than other domains of MCKRS (*r* < 0.510). Due to these reasons, the MCRKS (Per) was considered a valid outcome measure concerning the construct validity.

**Table 4:** Spearmen correlation coefficients between different domains of the modified Cincinnati knee rating system (MCRKS) and other knee scores.

MCRKS subscales	Pain	Swelling	Giving away	Activity level	Walking	Stairs	Running activity	Jumping	Total score
Lysholm									
* r*	0.720	0.551	0.502	0.519	0.631	0.703	0.639	0.615	0.794
* p*‐Value	<0.001	<0.001	<0.001	<0.001	<0.001	<0.001	<0.001	<0.001	<0.001
Tegner									
* r*	0.290	0.142	0.123	0.392	0.400	0.208	0.476	0.436	0.421
* p*‐Value	<0.003	0.156	0.220	<0.001	<0.001	0.037	<0.001	<0.001	<0.001
ACL‐RSI									
* r*	0.567	0.377	0.433	0.455	0.426	0.513	0.563	0.562	0.642
* p*‐Value	<0.001	<0.001	<0.001	<0.001	<0.001	<0.001	<0.001	<0.001	<0.001
SANE									
* r*	0.680	0.492	0.322	0.643	0.569	0.654	0.573	0.602	0.721
* p*‐Value	<0.001	<0.001	<0.001	<0.001	<0.001	<0.001	<0.001	<0.001	<0.001
HSS ACL‐SS									
* r*	0.653	0.455	0.480	0.567	0.540	0.621	0.682	0.718	0.782
* p*‐Value	<0.001	<0.001	<0.001	<0.001	<0.001	<0.001	<0.001	<0.001	<0.001
VAS pain									
* r*	−0.538	−0.331	−0.160	−0.342	−0.371	−0.517	−0.419	−0.392	−0.507
* p*‐Value	<0.001	0.001	0.112	0.001	<0.001	<0.001	<0.001	<0.001	<0.001

Abbreviations: ACL‐RSI, anterior cruciate ligament return to sport after injury; HSS ACL‐SS, hospital for special surgery ACL satisfaction; SANE, single assessment numeric evaluation; SD, standard deviation; VAS, visual analog scale.

## DISCUSSION

This investigation demonstrates the favourable measurement features of MCRKS (Per) with no challenges in terms of comprehension and cultural adaptability. Furthermore, this measure demonstrated both reliability and validity in assessing the clinical result and satisfaction of patients who had received ACL reconstruction surgery. As far as we know, the Arabic MCRKS is the sole scale that has been translated and modified across different cultures [29]. The findings of this study can be used as a foundation for future verifications and adaptations of MCRKS. The test‐retest reliability of the ‘swelling’ subscale ranged from an ICC of 0.82 to an ICC of 0.96 for the ‘giving away’ subscale. Nevertheless, the interclass correlations were found to be lower in the Italian version (0.76–0.91) and greater in the Brazilian–Portuguese version of MCKRS (0.96–0.99) [12, 42]. Furthermore, our scale demonstrated exceptional internal consistency, as evidenced by a Cronbach's *α* coefficient of 0.908. This is notably higher than the *α* values of 0.76 and 0.78 observed in the Italian and Brazilian CKRS, respectively. The construct validity, as measured by Pearson's correlation coefficient (*r*), ranged from 0.55 to 0.72 for the Lysholm score in the Brazilian CKRS. This correlation was found to be poor, ranging from 0.19 to 0.82. However, it was statistically significant for both the Brazilian and Italian versions (Table 5).

**Table 5:** Test‐retest reliability, internal consistency, and validity of the modified and original Cincinnati knee rating score in different languages.

Study and reference	Language	Population	Test‐retest reliability (ICC)	Cronbach's *α*	Validation scores	Correlation (Spearman's *r*)
Khaja 2020 MCRKS [28]	Arabic	57	>0.70	0.792	KOOS	0.76
Crespi 2020 CKRS [12]	Italian	124	0.85	0.76	Lysholm	0.75
Ramos Marinho 2020 CKRS [42]	Brazilian‐Portuguese	150	0.96–0.99	0.54–0.79	Lysholm	0.19–0.82
This study 2024	Persian	101	0.971	0.908	Lysholm	0.794
					Tegner	0.421
					HSS ACL‐SS	0.782
					ACL‐RSI	0.642
					VAS pain	−0.507
					SANE	0.721

Abbreviations: ACL‐RSI, anterior cruciate ligament return to sport after injury; HSS ACL‐SS, hospital for special surgery ACL satisfaction; SANE, single assessment numeric evaluation; SD, standard deviation; VAS, visual analog scale.

Each patient‐reported outcome measurement (PRO) has a unique pattern of coverage across many domains (such as pain, symptoms, functional activities, occupational activities, sport/recreation, and quality of life) when assessing knee‐specific ACL injuries. The HSS ACL‐SS comprises 10 items that assess the level of patient satisfaction following ACLR. Patients assign numerical values to each question, resulting in a minimum score of 10 out of 50 and a maximum score of 50 out of 50. The MCKRS scale comprises multiple items that are categorised into five categories, each with its own distinct scoring system. Superior ratings indicate improved clinical circumstances following ACL surgery [26]. The Lysholm and Tegner activity measures were originally developed to assess cruciate ligament injuries, particularly those involving the ACL [9, 30, 51]. These scales have extensive usage in evaluating knee function and activity levels after surgery [10, 23]. The Lysholm score is employed to assess the efficacy of the knee joint in different activities, including work, sports, and leisure. Furthermore, this scale functions as an additional instrument to the knee functional rating system, with a scoring range of 0–100 [51]. The Lysholm scale demonstrates a strong association with other scales that specifically assess the knee. On the other hand, the MCRKS has been proven to be a valid, reliable, and responsive tool for evaluating knee function and symptoms in patients who have suffered an ACL injury and undergone reconstruction [32]. The Tegner activity scale consists of a numerical scale ranging from 0 to 10. A score of 0 indicates persons who are unable to work or need disability benefits because of knee‐related problems, while a score of 10 represents individuals who participate in competitive sports at a relatively high level [50]. The Single Numeric Assessment [13] is a subjective rating system used to evaluate patients' results. It involves asking patients to rank their wounded body part on a scale ranging from 0 to 100 [56]. Consequently, patients may not be required to respond to several inquiries. Furthermore, there are reports indicating a strong correlation between the SANE score and knee assessment measures such as the IKDC score and the knee outcome survey (KOS) [45]. The visual analogue scale (VAS) is a reliable and highly reproducible subjective measure used to assess a patient's pain level on a scale of 0–10 [6, 27]. However, total score of our Persian version of mKCRS showed significant moderate correlation with other PROs. but, two subscales presented weak correlations with the Tegner activity score, for items ‘swelling’ (*r* = 0.14) and the ‘giving away’ (*r* = 0.12). Selbourne et al. [45] reported moderate strong correlation between SANE and MCRKS (*r* = 0.66) which was quite similar with our findings (*r* = 0.71). With regards to floor and ceiling effect, no relevant effect was present as in Arabic version of MCRKS.

This study should encourage rehabilitators worldwide to validate the test in their own cultural context so that it can become a reference measure in assessing clinical conditions of patients after ACLR surgery.

## LIMITATIONS

Our study is subject to several limitations. Initially, we did not assess the structural validity of the MCRKS (Per) instrument. Structural validity examines the dimensionality of the instrument and ensures that the subscales or domains within the instrument are distinct and measure different constructs. As per the COSMIN guidelines, structural validity is an important psychometric property that should be evaluated, especially in the early stages of validation studies. Future research should include a comprehensive assessment of structural validity, such as factor analysis or structural equation modelling, to confirm the hypothesised factor structure and dimensionality of the MCRKS (Per). Second, participants completed the questionnaire just once, hence this study does not provide any information regarding the responsiveness of MCRKS (Per). Third, we did not include patients with recurrent ACL injury or patients with ACL defect. It is advisable to utilise this scale for the evaluation of various types of ACL injuries beyond its original intended use. fourth, our study did not include any control group, so we were not able to show discriminant validity of our scale.

## CONCLUSION

The present study gives evidence about the reliability and validity of MCRKS (Per) for health professionals and researchers. The MCRKS can be considered suitable for the use in the Iranian population for the assessment and monitoring of ACL reconstruction recovery.

## AUTHOR CONTRIBUTIONS

Peyman Mirghaderi and Nasim Eshraghi contributed to the study conception and design, analysed data and edited the manuscript. Reza Omid contributed to the study design and wrote the first draft of the manuscript. Mohamad Sajadi and Amirreza Pashapour‐Yeganeh contributed to the study design, data collection and drew figures. S. M. Javad Mortazavi supervised the project, validate data and revised the manuscript. All authors commented on previous versions of the manuscript and revised it. All authors read and approved the final manuscript.

## CONFLICT OF INTEREST STATEMENT

The authors declare no conflict of interest.

## ETHICS STATEMENT

The study was reviewed and approved by the Institutional Review Board of Tehran University of Medical Sciences. An informed consent signed by all the participants of study. Patient consent was obtained regarding publication of data and photographs.

## Data Availability

The data that support the findings of this study are available from the corresponding author, S. M. Javad Mortazavi, upon reasonable request.
